# 2OM-Pred: prediction of 2-*O*-methylation sites in ribonucleic acid using diverse classifiers

**DOI:** 10.1093/bib/bbaf282

**Published:** 2025-06-17

**Authors:** Anas Bilal, Muhammad Taseer Suleman, Khalid Almohammadi, Abdulkareem Alzahrani, Xiaowen Liu

**Affiliations:** Department of Information Science and Technology, Hainan Normal University, No. 99 Long Kun South Road, Haikou 571158, China; Department of Computer Science, Bahria University Lahore Campus, 47-Civic Centre, Johar Town, Lahore, Punjab 54782, Pakistan; Computer Science Department, Applied College, University of Tabuk, King Faisal Road, Tabuk City 71491, Tabuk Region, Saudi Arabia; Computer Science Department, Faculty of Computing and Information, Al-Baha University, King Fahad Road, Alaqiq 65779, Al-Baha Region, Saudi Arabia; Department of Information Science and Technology, Hainan Normal University, No. 99 Long Kun South Road, Haikou 571158, China

**Keywords:** genomics, RNA, PTM, 2OM, transcriptomics

## Abstract

2-*O*-methylation (2OM) is a vital post-transcriptional modification which is formed by a functional group through the attachment of a methyl (–CH3) group to the second position of an aromatic ring hydroxyl group (–OH). It plays an active part in RNA physical configuration stability and the way different RNA molecules interrelate. Further, this modification plays a pivotal role in changing the epigenetic regulation of cellular processes. Previous approaches like mass spectrometry could not fully enhance the identification of RNA-modified sites. Sequence data were useful in the development of measures that meant the use of computationally intelligent system to identify 2OM sites quickly. This research proposed a new novel method of feature extraction and generation from the available sequences, and the feature dimensionality reduction has been done through the incorporation of statistical moments. The final feature vectors were developed and used to train prediction models. The assessment of prediction models was carried out through independent set tests and *k*-fold cross-validation. Through rigorous testing, the bagging ensemble model outperformed and revealed optimal accuracy scores. A publicly accessible web-based application has been developed which can be accessed via https://2om-pred-webapp.streamlit.app/.

## Introduction

**Figure 1:**
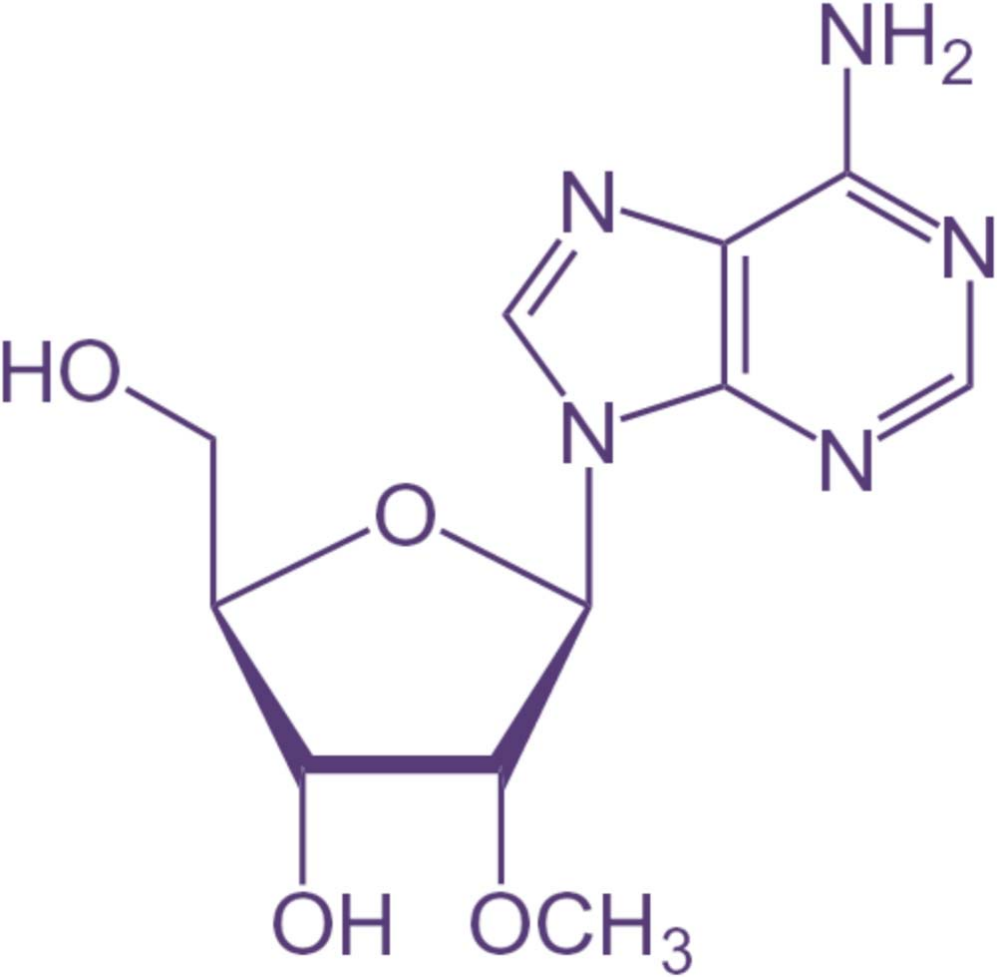
2OM chemical structure.

Post-transcriptional modification (PTM) in ribonucleic acid (RNA) describes some alteration that can be made to an RNA molecule once it has already been transcribed from DNA [1–3]. This means that many changes are possible depending on the RNA, including additions, deletions, insertions, rearrangements, and even changes in the molecule’s behavior in RNA. PTMs incorporate capping, splicing, methylation, polyadenylation, along with modifications to bases that ultimately contribute to the RNA translation into proteins. So far, over a hundred PTMs have been identified, of which 2-*O*-methylation (2OM) is among the extremely prevalent modifications [4]. 2OM is a derivative in which a methyl (CH3) group is attached to the second-position hydroxyl (OH) group of a molecule [5, 6]. In biological systems, methylation is a frequent chemical alteration that can significantly alter the molecule’s function. 2OM is commonly linked to RNA in biochemistry [7, 8]. The transmission of genetic information from DNA to protein synthesis is facilitated by RNA, a molecule with a pivotal function in the process [9]. The modification is linked to a few diseases, such as non-syndromic X-linked mental retardation [10] and diabetes II [11, 12]. Different impacts on RNA stability, structure, and function can result from methylation of the 2′-OH, the sugar component of the RNA molecule like ribose [13, 14]. For instance, 2OM can inhibit ribonuclease-mediated RNA degradation, modify RNA folding and secondary structure, and control RNA–protein interactions [15, 16]. Figure 1 represents the chemical structure of 2OM.

Recent studies have explored diverse aspects of molecular biology and computational genomics, including microRNA (miRNA) regulation in allergens [17], N7-methylguanosine prediction using deep learning [18], and the role of m6A modification in plant development [19, 20]. Additionally, research has investigated miRNA-mediated tumor heterogeneity [21], belief shifts clustering in computational systems [22], and tobacco smoke-induced craniofacial deformities [23]. Other work has examined lncRNA interactions in angiogenesis [24], immune infiltrate risk factors in cancer [25], methylation models in gastric cancer [26], and continual learning approaches in artificial intelligence [27]. Moreover, Pham *et al*. [28] developed a deep learning-based 2OM predictor, H2O-Pred, for four different nucleotides, including Guanine (G), Cytosine (C), Adenine (A), and Uracil (U). The proposed system is a stacked-based multi-model system that incorporates Natural Language Processing (NLP) based methods. Yang *et al*. [29] Proposed an support vector machine (SVM)-based iRNA-2OM for detecting Homosapien’s sites in 2OM. The model was trained using 147 positive 2OM sites and 147 negative 2OM sites. All RNA samples were 41-nt long, and the central nucleotide was methylated. Similarly, five-fold cross-validation was used to evaluate the model’s performance, and the proposed strategy achieved 97.95% accuracy. Li *et al*. [30] established a webserver, DeepOMe, a deep-learning-based predictor to predict 2OM sites in human messenger RNA (mRNA). The training, along with testing data samples for the proposed model, comprised 4481 2OM sites related to the Homosapiens transcriptome. All RNA samples were 290-nt long. The DeepOMe revealed a 97.5% accuracy (ACC) score. Mostavi *et al*. [31] used 147 positive and negative 2OM samples for training the proposed model, deep-2′-*O*-Me, based on Convolutional Neural Network (CNN). The model revealed 0.89 Area Under the Curve (AUC) and 0.88 Area Under the Precision–Recall Curve (auPRC) values.

The present research aimed to enhance computational models’ ability to identify 2OM sites by developing novel feature extraction techniques. A range of ensemble learning models was designed and evaluated, utilizing vectors incorporating both positional and compositional information. The dataset used for the study was compiled from prior research, allowing for a meaningful comparison with existing models. Key features considered during model development included the compositional makeup of nucleotide sequences, the relative and absolute positions of nucleotides, and the frequency of occurrence for each nucleotide. Independent set tests and *k*-fold cross-validation were employed to validate the robustness and reliability of the models. The results demonstrated that the newly developed model, 2OM-Pred, outperformed other 2OM site predictors in various evaluation metrics. Importantly, the same dataset was used across different tests, ensuring a consistent basis for comparison. This research implies that it will help boost the identification process of 2OM sites in RNA, which is time-consuming and difficult in traditional laboratory methods. 2OM sites are found abundantly in transfer RNA, mRNA, and miRNA and are also linked to disease pathogenesis. Therefore, with the availability of sequence data, it is possible to build such computationally intelligent models to optimize the identification of 2OM sites within RNA.

To ensure applicability in real-life scenarios, particularly for researchers without computational expertise, the elaboration has been made on how the model can be effectively utilized. The proposed method leverages position-related features by encoding input sequences centered around putative or experimentally validated methylation sites. In practical terms, this means that for prediction tasks, the input to the model should be a sequence window (e.g. ±*X* nucleotides) around a target site. Since fixed-length sequences have been used with size 41 bp, the central nucleotide has positioned at 21. A web-based tool has been provided to the research community for validation, accessed via https://2om-pred-webapp.streamlit.app/. Biologists can provide genomic sequences of any length. The web-based tool scans the sequence, extracts fixed-length windows centered around candidate sites, and prepares them in the format required by the model. Once the centered sequences are generated, they can be fed directly into the trained model. The model then revealed prediction results on whether such a site is 2OM or non-2OM. These predictions can be mapped back onto the genome, allowing researchers to visualize methylation predictions in genomic browsers or integrate them with transcriptomic, epigenomic, or phenotypic data.

## Methodological framework

### Datasets collection

**Figure 2:**
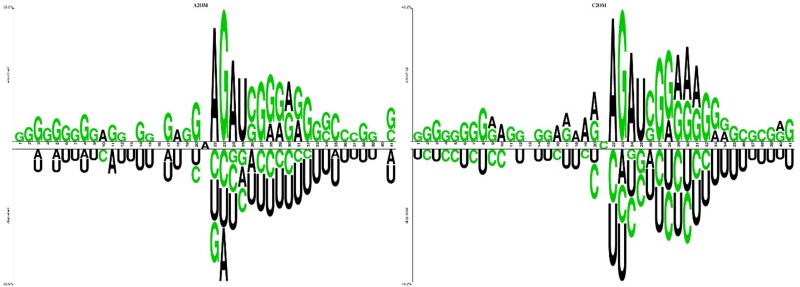
Two sample logos for A2OM and C2OM depicting nucleotide distribution within sequences.

For the existing research, the H2O-Pred [28] dataset has been considered for experimentation purposes. The dataset serves as the benchmark collection for research, containing no duplicate entries and outperforming all existing public datasets. The dataset was refined, and samples can be validated in widely used databases such as RMBase [32]. The dataset contained significant samples of positive (2OM sites) and negative (non-2OM sites) classes. In total, 7597 2OM sites were gathered from various genomic regions and contexts. The prepared dataset was divided into four sets corresponding to G, C, U, and A for G2OM, C2OM, U2OM, and A2OM, respectively. Table 1 indicates the sample details of each nucleotide-specific 2OM site. A web-based two-sample logo [33] has been used to generate a web logo of sequence data. These web logos helped visualize the data distribution of nucleotides. As can be observed in Fig. 2, there is a concentration of "G" in the second half of the nucleotide sequences in the enriched region of both A2OM and C2OM datasets.

**Table 1:** Sample details of each nucleotide-specific 2OM site

**2OM site**	**Training samples**	**Testing samples**
A2OM	2176	934
C2OM	2278	788
G2OM	1832	788
U2OM	2236	960

**Figure 3:**
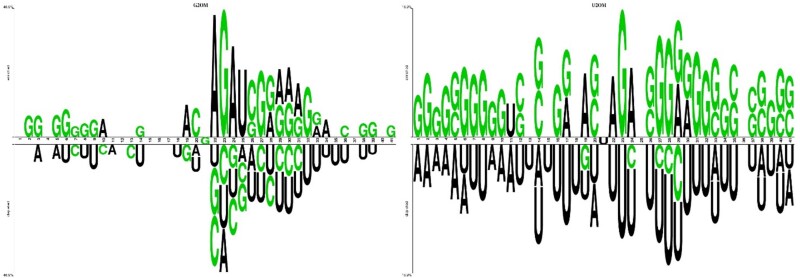
Two sample logos for G2OM and U2OM depicting nucleotide distribution within sequences.

Similarly, a concentration of "G" and "U" can be observed in Fig. 3 in both enriched and depleted regions, respectively, for G2OM. However, nucleotides have been evenly distributed in U2OM, as shown in Fig. 3.

**Figure 4:**
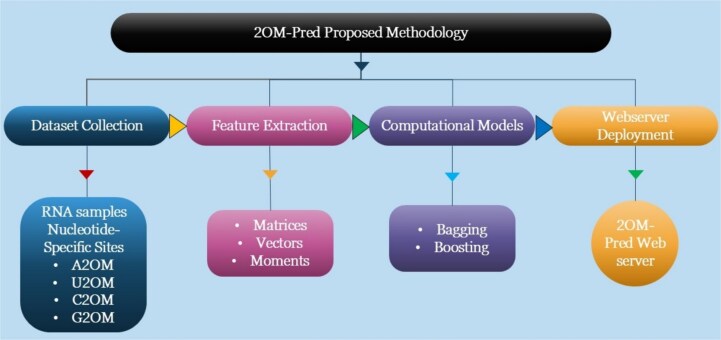
2OM-Pred methodology flow diagram.

Notably, in the current research, similar training samples have also been used for computational models’ development and training. Figure 4 depicts the proposed methodology of the 2OM-Pred model. The RNA samples from four different nucleotide-specific sites were utilized for feature extraction. A novel feature generation mechanism has been proposed in the research. The features obtained were then subjected to ensemble model training and validation as well. Finally, a webserver was developed to facilitate the research community’s access to 2OM-Pred.

### Features development model

**Figure 5:**
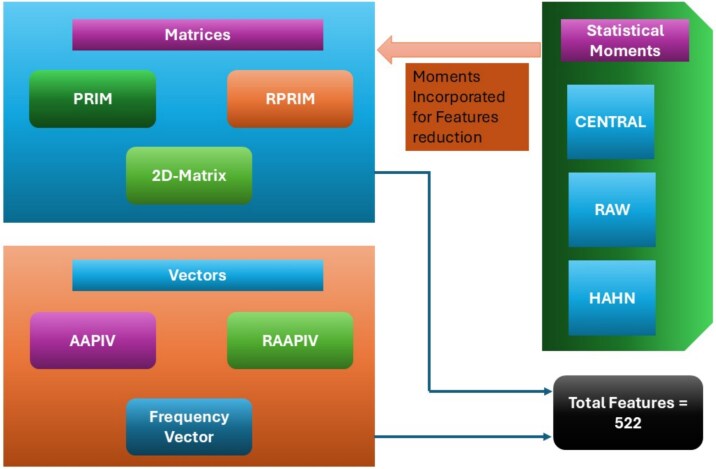
Feature extraction methodology flow chart depicting matrices and vectors.

The transformation of biological sequence data into numerical vectors helped provide input to the computational models. For the current research study, few models have been developed to effectively capture clearly observable and hidden patterns within RNA sequences [34, 35]. Figure 5 presents a flow chart of the devised feature extraction model, including various matrices and vectors employed for feature extraction from the given biological sequences.

The sequence arrangement and content of nucleotides were analyzed to create feature vectors. Several matrices and vectors were used to help attain attributes from the available RNA sequences. Chou first implemented this idea in the form of pseudo amino acid composition, which is used for protein data [36, 37]. A unique feature extraction model has been proposed based on Chou’s proposed methodology, which has helped train prediction models [38, 39]. The RNA samples in the dataset have been formulated as provided in Equation (1):


(1)

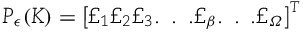



The expression represents each nucleotide, 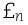, in a given sequence. The central nucleotide, 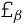, denotes the 2OM site. The transpose function, *T*, was applied to the formulation of the accumulated features. Moreover, for the current research study, 41 bp nucleotide sequence length has been considered for obtaining optimal results in terms of prediction accuracy. Equation (2) describes the sequence length:


(2) \begin{equation*} P={R}_1{R}_2{R}_3\cdots{R}_{18}{R}_{19}{\boldsymbol{R}}_{\mathbf{21}}\cdots{R}_{39}{R}_{40}{R}_{41} \end{equation*}

The term, ${\boldsymbol{R}}_{\mathbf{21}}$, in Equation (2) presents the central nucleotide to be guanine, adenine, cytosine, and uracil.

Statistical moments capture all essential features of a dataset, including its distribution shape, spread, and central measures and thus used for feature dimensionality reduction [40]. We calculated Hahn, Central, and Raw moments for this research study. Raw and Hahn moment statistics depend on dataset scaling and the relative positioning of values. Central moments vary based on scaling and position. Hahn moments are calculated using Hahn polynomials, which are used to compute Hahn moments to preserve sequence positioning data. These moments were employed to simplify the output from matrix models. The matrix ${\mathrm{Q}}^{\prime }$ in Equation (3) is a two-dimensional array with *x* * *y* cells, where ${q}_{mn}$ represents the $n\mathrm{th}$ nucleotide base in the $m\mathrm{th}$ sequence:


(3) \begin{equation*} {\mathrm{Q}}^{\prime }=\left[\begin{array}{@{}cccc@{}}{q}_{11}& {q}_{12}& \dots & {q}_{1n}\\{}{q}_{21}& {q}_{22}& \dots & {q}_{2n}\\{}\vdots & \vdots & \ddots & \vdots \\{}{q}_{m1}& {q}_{m2}& \dots & {q}_{mn}\end{array}\right] \end{equation*}

Variance, mean, and unequal probability distribution of data samples were calculated using raw moments. Raw moments expressed in Equation (4) where $u+v$ is the summation of raw moments and ${L}_{00}$, ${L}_{01}$, ${L}_{10}$, ${L}_{11}$, ${L}_{12}$, ${L}_{21}$, ${L}_{30}$, ${L}_{03}$, were calculated up to a third-degree polynomial. Raw moment expressed in Equation (4) represents the probability distribution of components:


(4) \begin{equation*} {L}_{uv}=\sum_{a=1}^m\sum_{b=1}^m{a}^u{b}^v{\beta}_{ab} \end{equation*}

Central moments are location-independent and more correlated with distributions’ composition and shape. Central moments were computed based on the deviations from the data mean value. The central moments were calculated for the existing research as stated in Equation (5):


(5) \begin{equation*} {n}_{ij}=\sum_{b=1}^n\sum_{q=1}^n{\left(b-x\right)}^i{\left(q-y\right)}^j{\beta}_{bq} \end{equation*}

Hahn moments [41] were determined using Hahn-polynomials, as presented in Equation (6):


(6) \begin{align*} &{h}_n^{u,v}\left(r,N\right)={\left(N+V-1\right)}_n{\left(N-1\right)}_n\times \sum_{k=0}^n{\left(-1\right)}^k\nonumber \\&\qquad \frac{{\left(-n\right)}_k\ast{\left(-r\right)}_k\ast{\left(2N+u+v-n-1\right)}_k}{{\left(N+v-1\right)}_k\ast{\left(N-1\right)}_k}\ast \frac{1}{k!} \end{align*}

In order to compute normalized orthogonal Hahn moments of the two-dimensional data, Equation (7) has been used. The computation of Hahn moments is essential as it represents an accurate data analysis:


(7) \begin{equation*} {H}_{ij}=\sum_{q=0}^{N-1}\sum_{p=0}^{N-1}{\beta}_{ij}{h}_j^{\widetilde{u,v}}\left(q,N\right){h}_j^{\widetilde{u,v}}\left(p,N\right),\kern0.5em m,n=0,1,\dots N-1 \end{equation*}

Incorporating these statistical moments into the matrix generated a relatively large number of values. So, through these moments, only significant values were extracted from matrices that would contribute to ensemble model training in an optimized manner.

The relative position of each nucleotide with other nucleotides reveals critical information about the patterns within RNA sequences. To extract this information, three combinations of nucleotides have been considered: mononucleotide, dinucleotide, and trinucleotide. These developed matrices were used to uncover the relative positions of nucleotide bases, which aided in systematically quantizing the relative positions of nucleotides. A general form of these three matrices has been represented in Equation (8) as ${G}_{\mathrm{PRIM}}$:


(8) \begin{equation*} {G}_{\mathrm{PRIM}}=\left[\begin{array}{@{}ccccccc@{}}{G}_{1\to 1}& {G}_{1\to 2}& {G}_{1\to 3}& \dots & {G}_{1\to y}& \dots & {G}_{1\to j}\\{}{G}_{2\to 1}& {G}_{2\to 2}& {G}_{2\to 3}& \dots & {G}_{2\to y}& \dots & {G}_{2\to j}\\{}{G}_{3\to 1}& {G}_{3\to 2}& {G}_{3\to 3}& \dots & {G}_{3\to y}& \dots & {G}_{3\to j}\\{}\vdots & \vdots & \vdots & & \vdots & & \vdots \\{}{G}_{x\to 1}& {G}_{x\to 2}& {G}_{x\to 3}& \dots & {G}_{x\to y}& \dots & {G}_{4\to j}\\{}\vdots & \vdots & \vdots & & \vdots & & \vdots \\{}{G}_{N\to 1}& {G}_{N\to 2}& {G}_{N\to 3}& \dots & {G}_{N\to y}& \dots & {G}_{N\to j}\end{array}\right] \end{equation*}

More information can be extracted from the reversed sequence. For this purpose, a reverse ${K}_{\mathrm{PRIM}}$ matrix was developed, which was used to extract relative position information from the sequence in the opposite order, as shown in Equation (9):


(9) \begin{equation*} {K}_{\mathrm{RPRIM}}=\left[\begin{array}{@{}ccccccc@{}}{K}_{1\to 1}& {K}_{1\to 2}& {K}_{1\to 3}& \dots & {K}_{1\to y}& \dots & {K}_{1\to j}\\{}{K}_{2\to 1}& {K}_{2\to 2}& {K}_{2\to 3}& \dots & {K}_{2\to y}& \dots & {K}_{2\to j}\\{}{K}_{3\to 1}& {K}_{3\to 2}& {K}_{3\to 3}& \dots & {K}_{3\to y}& \dots & {K}_{3\to j}\\{}\vdots & \vdots & \vdots & & \vdots & & \vdots \\{}{K}_{x\to 1}& {K}_{x\to 2}& {K}_{x\to 3}& \dots & {K}_{x\to y}& \dots & {K}_{4\to j}\\{}\vdots & \vdots & \vdots & & \vdots & & \vdots \\{}{K}_{N\to 1}& {K}_{N\to 2}& {K}_{N\to 3}& \dots & {K}_{N\to y}& \dots & {K}_{N\to j}\end{array}\right] \end{equation*}

The frequency count of each nucleotide is the occurrence of a typical nucleotide with RNA sequences. For the current research, frequency vector *f*′ has been provided in Equation (10), which provides nucleotide within the available RNA sequence.


(10) \begin{equation*} {f}^{\prime }=\left\{{\mu}_1,{\mu}_2,\dots ..,{\mu}_n\right\} \end{equation*}

The PRIM and RPRIM matrices only provide information about the relative position of nucleotides in both normal and reverse order. Moreover, accumulative positional information of nucleotides within RNA sequences was extracted through vectors ${C}_{\mathrm{AAPIV}4}$, ${C}_{\mathrm{AAPIV}16}$, ${C}_{\mathrm{AAPIV}64}$ in Equations (11), (12), and (13) respectively:


(11) \begin{equation*} {C}_{\mathrm{AAPIV}4}=\left\{{\rho}_{1,}{\rho}_{2,}{\rho}_{3,}{\rho}_{4,}\right\} \end{equation*}

(12) \begin{equation*} {C}_{\mathrm{AAPIV}16}=\left\{{\rho}_{1,}{\rho}_{2,}{\rho}_{3,}\dots, {\rho}_{15,}{\rho}_{16,}\right\} \end{equation*}

(13) \begin{equation*} {C}_{\mathrm{AAPIV}64}=\left\{{\rho}_{1,}{\rho}_{2,}{\rho}_{3,}\dots, {\rho}_{63,}{\rho}_{64}\right\} \end{equation*}

Any component ${\exists}_i$ is calculated as expressed in Equation (14):


(14) \begin{equation*} {\exists}_i=\sum_{k=1}^n{\exists}_k \end{equation*}

By reversing the sequence, more information about positions can be extracted from the RNA sequences. For this purpose, a reverse accumulative absolute position incidence vector $\left(\mathrm{NRAAPIV}\right)$ has been formed, which is represented ${N}_{\mathrm{RAAPIV}4}$ Equation (15), ${N}_{\mathrm{RAAPIV}16}$ Equation (16), and ${N}_{\mathrm{RAAPIV}64}$ Equation (17), respectively:


(15) \begin{equation*} {N}_{\mathrm{RAAPIV}4}=\left\{{\tau}_{1,}{\tau}_{2,}{\tau}_{3,}{\tau}_4\right\} \end{equation*}

(16) \begin{equation*} {N}_{\mathrm{RAAPIV}16}=\left\{{\tau}_{1,}{\tau}_{2,}{\tau}_{3,}\dots, {\tau}_{16}\right\} \end{equation*}

(17) \begin{equation*} {N}_{\mathrm{RAAPIV}64}=\left\{{\tau}_{1,}{\tau}_{2,}{\tau}_{3,}\dots, {\tau}_{64}\right\} \end{equation*}

The final feature extraction phase is the formulation of a feature vector, which contains 522 distinct values derived from matrices and vectors. Statistical moments were incorporated into PRIM and RPRIM to reduce values obtained from these matrices. In this way, the dimension of features was reduced to 522, which helped develop robust models. Table 2 contains the values obtained from various vectors and matrices employed in this research.

**Table 2:** Number of features obtained from matrices and vectors

**Vector/matrix**	**No. of features**
PRIM matrices (post features dimensionality reduction)	90
RPRIM matrices (post features dimensionality reduction)	90
Frequency vector	84
AAPIV vectors	84
RAAPIV vectors	84
2D matrix	90
Total	522

The features used to train ensemble models were derived from a comprehensive set of sequence descriptors, which underwent dimensionality reduction by incorporating statistical moment techniques. Specifically, feature values were extracted from matrices such as PRIM and RPRIM, which capture position-specific nucleotide distributions and sequence dependencies. We retained essential information by applying moment-based feature reduction to these matrices while minimizing redundancy. In addition to these matrices, the 2D matrix has been utilized to encode higher-order sequence relationships, along with several important feature vectors, including frequency vector (FV), AAPIV, and RAAPIV. These form a unique feature set of 522 reduced features, which contributed to optimizing the prediction performance.

These features were incorporated into the resultant feature vectors due to their unique contribution toward the features set, which in turn is used for training computationally intelligent models, i.e. Ensemble models in this research study. Integrating these diverse features significantly contributed to the robustness and accuracy of the models in predicting 2OM sites.

The final feature vector was obtained from FV, AAPIV, RAAPIV, PRIM, and RPRIM. Since the problem in this research addressed binary classification, the positive sequences were labeled as "1" and the negative sequences as "0." These feature vectors were used to train prediction models.

### Ensemble models incorporation

**Figure 6:**
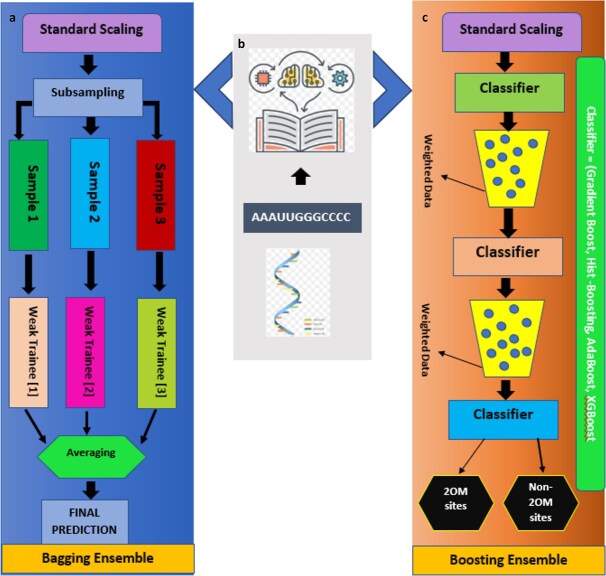
(a) Bagging ensemble models. (b) RNA samples input with feature extraction model. (c) Boosting ensemble models.

Ensemble learning techniques have garnered significant optimism in addressing complex machine learning problems due to their superior performance compared to single-model approaches. Modern ensemble models are classified into parallel and sequential methods, which combine different model types to achieve better results. Ensemble methods address real-world problems by improving accuracy by merging predictions and discovering different patterns through feature observation. Multiple trained models work in parallel when bootstrapping or bagging is used, where parts of the training dataset are employed for each model. In contrast, sequential strategies train models one after another, with each model adopting itself from the errors made by the previous one. Research continually explores the use of ensemble methods for data classification. Figure 6 represents the ensemble models incorporated in this research. For bagging ensemble models, various algorithms were incorporated, including random forest (RF), extra tree, decision tree (DT), and bagging ensemble. Moreover, XGBoost, AdaBoost, Gradient Boost, and Histo-gram-based boosting algorithms were used as ensemble boosting.

## Results and discussions

For the evaluation of prediction models, a few accuracy metrics were considered, which includes sensitivity (${S}_{ne}$), specificity (${S}_{pc}$), accuracy ($Acc$), and Matthews correlation coefficient (MCC) [42, 43]. It can also be noted that accuracy metrics were used to make a fair comparison with the comparative model i.e. H2O-Pred. False positive rate along with true positive rate, particularly ${S}_{pc}$, play a crucial role in the performance evaluation of binary classification models. ${S}_{pc}$ evaluates the model’s capability to identify true negative (TN) cases accurately. ${S}_{ne}$, on the other hand, evaluates the performance of a binary classifier when the positive class is of critical importance. The MCC is a robust metric often employed in binary classification, especially when dealing with imbalanced datasets. It takes into account all four components of the confusion matrix: true positive, TN, false positive (FP), and false negative (FN), offering a balanced evaluation of the model’s performance. Each accuracy metric used in this research study has been formulated and represented in Equation (18):


(18) \begin{equation*} \left\{\begin{array}{@{}c}{S}_{ne}=\displaystyle\frac{\mathrm{TP}}{\mathrm{TP}+\mathrm{FN}}\ 0\le{S}_{ne}\le 1\\[5pt] {}{S}_{pc}=\displaystyle\frac{\mathrm{TN}}{\mathrm{TN}+\mathrm{FP}}\kern1.5em 0\le{S}_{pc}\le 1\\[5pt] {} Acc=\displaystyle\frac{\mathrm{TN}+\mathrm{TP}}{\mathrm{TN}+\mathrm{FN}+\mathrm{TP}+\mathrm{FP}}\kern1.75em 0\le Acc\le 1\kern2.5em \\[5pt] {}\mathrm{MCC}=\displaystyle\frac{\left(\mathrm{TN}.\mathrm{TP}\right)-\left(\mathrm{FP}.\mathrm{FN}\right)}{\sqrt{\left(\mathrm{TP}+\mathrm{FN}\right)\left(\mathrm{TP}+\mathrm{FP}\right)\left(\mathrm{TN}+\mathrm{FN}\right)\left(\mathrm{TN}+\mathrm{FP}\right)}}-1\le \mathrm{MCC}\le 1\end{array}\right. \end{equation*}

The actual 2OM sites are labeled as TP, while non-2OM sites are labeled as TN. The label FN represents real 2OM locations that our system fails to detect, while FP indicates instances where the system incorrectly identifies something as a 2OM site. These metrics are relevant when the system handles a single classification task. Precise values for FP and FN are necessary to evaluate our system’s effectiveness. Errors in classification can lead to incorrect detection of 2OM sites within an RNA sample, resulting in FPs. Similarly, reducing classification stringency increases the risk of erroneously tagging additional non-2OM sites as 2OM sites.

### Dataset pre-processing

The feature set was pre-processed using standard scaling from the sci-kit learn library. During this process, the dataset was cleaned, and any missing values were handled, ensuring that the data were appropriately prepared for input into the machine learning.

### Independent testing set

**Figure 7:**
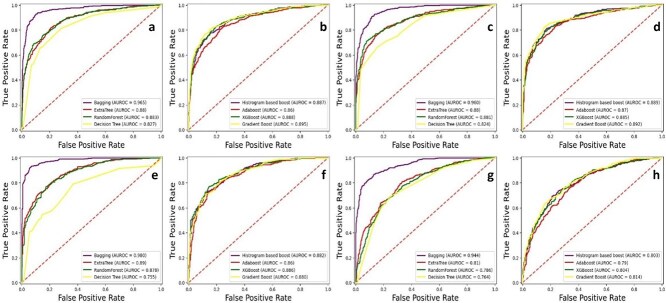
Independent set testing ROC graph for (a) A2OM bagging. (b) A2OM boosting. (c) C2OM bagging. (d) C2OM boosting. (e) G2OM bagging. (f) G2OM boosting. (g) U2OM bagging. (h) U2OM boosting.

Tests were conducted on independent datasets containing samples from A2OM, C2OM, G2OM, and U2OM, with results presented in tabular form. The samples were initially divided into an 80:20 ratio. The performance metrics for bagging as well as boosting ensemble models are shown in Table 3 for each dataset. The receiver operating characteristic (ROC) curve, displayed in Fig. 7, illustrates how effectively the binary model distinguishes each sample set.

**Table 3:** Independent set test for A2OM, C2OM, U2OM, and G2OM

Techniques		A2OM				G2OM				C2OM				U2OM			
		***A***_***CC***_	***S***_***pc***_	***S***_***en***_	***M***_***CC***_	***A***_***CC***_	***S***_***pc***_	***S***_***en***_	***M***_***CC***_	***A***_***CC***_	***S***_***pc***_	***S***_***en***_	***M***_***CC***_	***A***_***CC***_	***S***_***pc***_	***S***_***en***_	***M***_***CC***_
Bagging	Random forest	0.80	0.83	0.76	0.60	0.79	0.83	0.75	0.58	0.80	0.82	0.78	0.61	0.71	0.68	0.75	0.43
	Extra tree	0.80	0.80	0.81	0.60	0.79	0.80	0.78	0.59	0.80	0.82	0.79	0.61	0.73	0.68	0.78	0.47
	Decision tree	0.76	0.72	0.80	0.52	0.68	0.74	0.62	0.37	0.74	0.77	0.71	0.48	0.71	0.77	0.66	0.43
	Bagging	0.93	0.87	0.94	0.81	0.92	0.95	0.90	0.85	0.88	0.91	0.86	0.77	0.85	0.81	0.89	0.71
Boosting	Gradient boost	0.82	0.85	0.78	0.63	0.78	0.82	0.74	0.57	0.83	0.84	0.83	0.66	0.71	0.67	0.75	0.42
	Hist gradient boost	0.81	0.78	0.79	0.62	0.78	0.81	0.76	0.57	0.82	0.83	0.81	0.64	0.73	0.70	0.76	0.46
	Ada boost	0.78	0.77	0.85	0.57	0.78	0.80	0.77	0.57	0.79	0.80	0.78	0.58	0.73	0.70	0.76	0.46
	XGBoost	0.81	0.85	0.78	0.63	0.81	0.85	0.77	0.63	0.81	0.83	0.79	0.62	0.72	0.74	0.72	0.46

**Figure 8:**
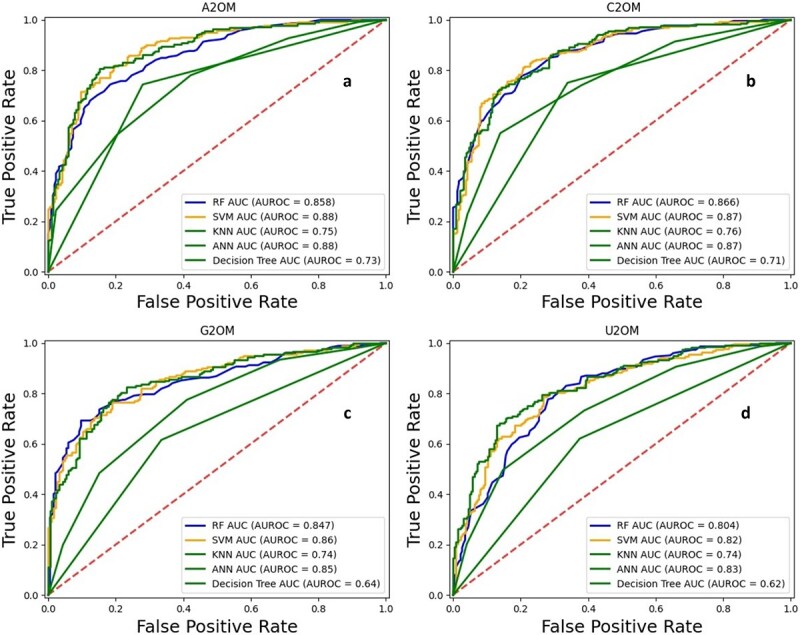
Independent testing set ROC graph of conventional machine learning models for (a) A2OM. (b) C2OM. (c) G2OM. (d) U2OM.

For cross-comparison, conventional machine learning models were also employed to validate the performance of ensemble models. Therefore, several models, including RF, SVM, *K*-nearest neighbor, artificial neural network, and DT, were used. The results of independent testing have been presented in Table 4. Moreover, Fig. 8 depicts the ROC graph of conventional machine learning algorithms.

**Table 4:** Independent set test for A2OM, C2OM, U2OM, and G2OM.

Models	A2OM				G2OM				C2OM				U2OM			
	***A***_***CC***_	***S***_***pc***_	***S***_***en***_	***M***_***CC***_	***A***_***CC***_	***S***_***pc***_	***S***_***en***_	***M***_***CC***_	***A***_***CC***_	***S***_***pc***_	***S***_***en***_	***M***_***CC***_	***A***_***CC***_	***S***_***pc***_	***S***_***en***_	***M***_***CC***_
RF	0.77	0.80	0.74	0.55	0.79	0.84	0.73	0.58	0.78	0.80	0.75	0.56	0.74	0.71	0.77	0.49
SVM	0.80	0.81	0.79	0.61	0.77	0.78	0.76	0.55	0.79	0.79	0.79	0.58	0.73	0.74	0.73	0.47
KNN	0.67	0.57	0.77	0.36	0.68	0.58	0.77	0.37	0.67	0.61	0.74	0.36	0.67	0.61	0.73	0.34
ANN	0.81	0.82	0.81	0.63	0.79	0.79	0.78	0.58	0.77	0.78	0.76	0.55	0.76	0.78	0.74	0.52
Decision tree	0.73	0.72	0.74	0.46	0.64	0.66	0.61	0.28	0.70	0.66	0.75	0.41	0.62	0.62	0.62	0.24

**Figure 9:**
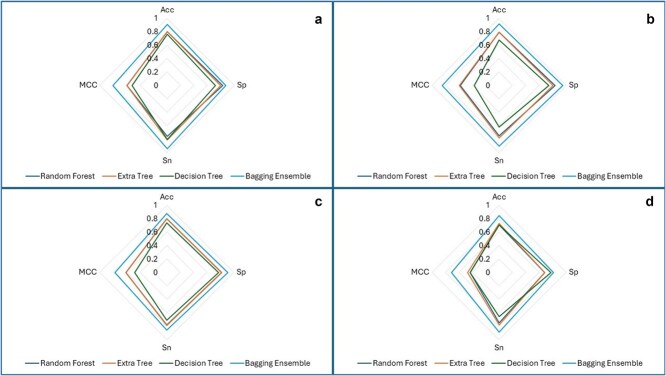
Spider charts of bagging ensemble models using independent set test for (a) A2OM. (b) G2OM. (c) C2OM. (d) U2OM.

**Figure 10:**
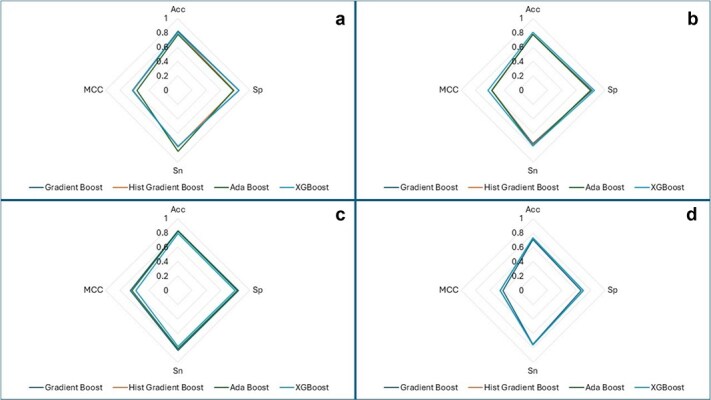
Spider charts of boosting ensemble models using independent set test for (a) A2OM. (b) G2OM. (c) C2OM. (d) U2OM.

Web charts, also known as spider graphs, are used to represent multi-metrics values of different categories. For this purpose, spider graphs have been drawn for each bagging ensemble model for each nucleotide-specific data used in this research. Figures 9 and 10 depict spider graphs obtained for independent set tests for bagging and boosting ensemble models, respectively.

### Cross-validation using *k*-fold

The cross-validation method analyzes all available data at once to keep results reliable. Our modeling process splits the data into k sections, where we use *k* − 1 sections for training as well as one part for testing. By setting *k* to 5, the study separated its dataset into five distinct sections. For every validation test round, four blocks of data acted as training data, and the remaining block functions as the test data. The model runs five distinct tests using each different part of the entire dataset to generate complete performance results. The results from five-fold cross-validation (CV) tests appear in Table 5.

**Table 5:** *K*-fold cross-validation test for A2OM, C2OM, U2OM, and G2OM

Techniques		A2OM				G2OM				C2OM				U2OM			
		***A***_***CC***_	***S***_***pc***_	***S***_***en***_	***M***_***CC***_	***A***_***CC***_	***S***_***pc***_	***S***_***en***_	***M***_***CC***_	***A***_***CC***_	***S***_***pc***_	***S***_***en***_	***M***_***CC***_	***A***_***CC***_	***S***_***pc***_	***S***_***en***_	***M***_***CC***_
Bagging	Random forest	0.80	0.84	0.81	0.60	0.81	0.83	0.70	0.64	0.81	0.80	0.71	0.61	0.72	0.71	0.73	0.44
	Extra tree	0.76	0.78	0.71	0.52	0.75	0.85	0.64	0.50	0.76	0.83	0.68	0.52	0.63	0.72	0.55	0.30
	Decision tree	0.73	0.75	0.68	0.45	0.70	0.70	0.71	0.41	0.70	0.69	0.71	0.42	0.60	0.60	0.61	0.21
	Bagging	0.83	0.85	0.81	0.66	0.75	0.85	0.63	0.51	0.81	0.87	0.75	0.62	0.73	0.68	0.77	0.46
Boosting	Gradient boost	0.87	0.87	0.85	0.74	0.77	0.84	0.70	0.55	0.80	0.84	0.77	0.62	0.74	0.72	0.76	0.45
	Hist gradient boost	0.83	0.92	0.75	0.66	0.77	0.77	0.76	0.54	0.77	0.82	0.64	0.55	0.73	0.69	0.77	0.46
	Ada boost	0.81	0.82	0.79	0.62	0.80	0.87	0.73	0.61	0.77	0.79	0.75	0.55	0.70	0.68	0.71	0.40
	XGBoost	0.83	0.87	0.78	0.66	0.81	0.81	0.78	0.60	0.73	0.77	0.71	0.47	0.74	0.71	0.78	0.50

**Figure 11:**
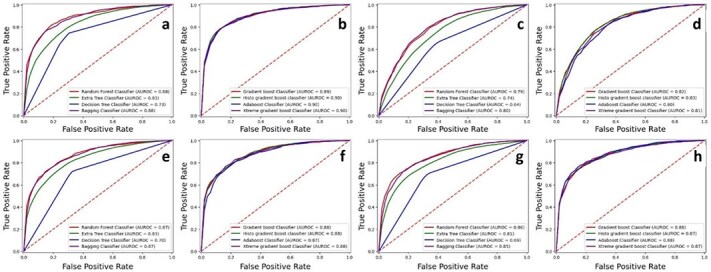
*K*-fold cross validation ROC graph for (a) A2OM bagging. (b) A2OM boosting. (c) U2OM bagging. (d) U2OM boosting. (e) C2OM bagging, (f) C2OM boosting, (g) G2OM bagging. (h) G2OM boosting.

Splitting the dataset into segments before running testing and training steps ensures better testing performance that matches real-world applications more accurately. A visual illustration highlights the results of validation performance tests in Fig. 11.

**Figure 12:**
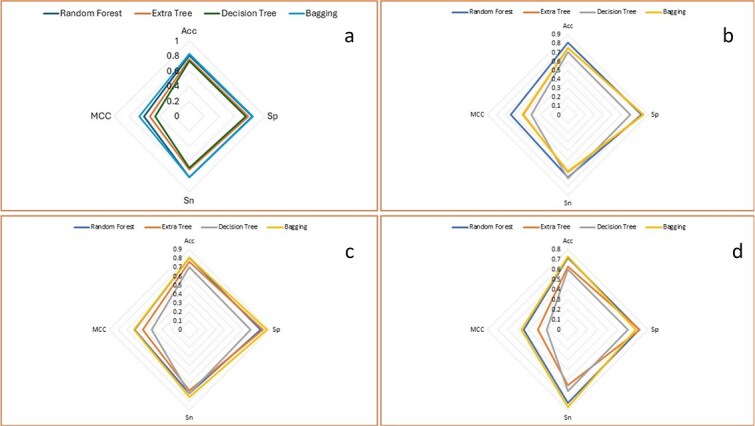
Spider charts of bagging ensemble models using *k*-fold cross-validation for (a) A2OM. (b) G2OM. (c) C2OM. (d) U2OM.

**Figure 13:**
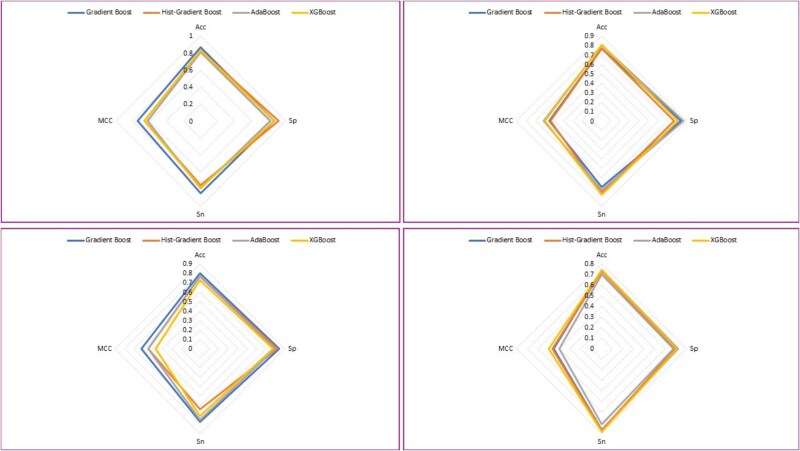
Spider charts of boosting ensemble models using *k*-fold cross-validation for (a) A2OM. (b) G2OM. (c) C2OM. (d) U2OM.

A spider graph of *k*-fold CV for both bagging along with boosting ensemble models is shown in Figs. 12 and 13, respectively.

**Figure 14:**
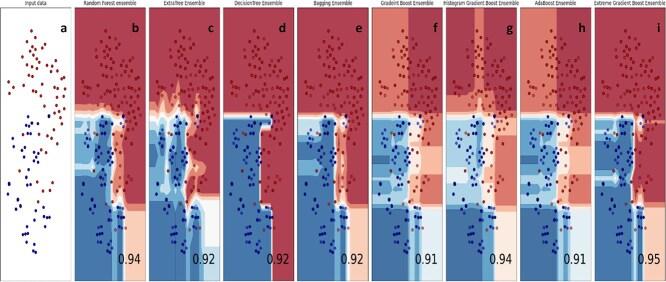
Boundary visualization of ensemble models. (a) Input. (b) Random forest. (c) Extra tree, (d) decision tree. (e) bagging. (f) Gradient boost. (g) histogram boost. (h) Adaboost. (i) Extreme gradient boost.

Supervised Machine Learning (ML) models for classification can be highly effective, but numerical predictions alone are sometimes insufficient. Visualizing the decision boundaries that separate different groups is crucial for a deeper understanding and improved classification accuracy. This study applied decision surface analysis to classification algorithms to enhance their accuracy. The decision surface map illustrates the model’s response across the input feature space. The model was initially trained using the training dataset. Once trained, the model was applied to predict outcomes for various input values. The decision surface was visualized using the contourf() function from Matplotlib, combined with a scatter plot to show data points. The decision-surface plots for the algorithms used for classification in this study are presented in Fig. 14.

Decision boundary visualization is a valuable tool used to illustrate how effectively a machine learning model can distinguish between different classes; in this case, binary classes represent positive (2OM sites) and negative (non-2OM sites) samples. This visualization provides intuitive insights into the model’s classification capabilities by showing how the decision surface separates the data points. In Fig. 14a, we observe a scatter plot composed of two distinct colors, each color representing one class of 2OM site samples: positive or negative. These colored dots indicate the distribution of the samples in the feature space, allowing us to assess how well-separated the two classes are visually. The subsequent subfigures within Fig. 14 depict the decision boundaries generated by various ensemble models used in this study. Each subfigure illustrates how a particular model classifies the samples by drawing the boundaries between the predicted classes. These visualizations help demonstrate the classification behavior of each model and highlight differences in their ability to identify and separate 2OM sites correctly.

This study established a reliable system for identifying 2OM sites by designing specialized feature extraction methods and developing and testing ensemble learning models. The 2OM is pivotal in regulating gene expression patterns during epigenetics, RNA processing, and stabilizing and rearranging mRNA. Moreover, this modification is also associated with a few diseases, such as intellectual disability syndrome and cancer. Traditional laboratory methods like mass spectrometry are efficient, but they are time-consuming processes. Moreover, the availability of RNA sequence data helped to develop indigenous computational models. The models were also subjected to rigorous testing and validation, through which more robust and reliable models could be developed to identify 2OM sites. The quick and accurate identification of 2OM sites helped us understand complex biological modifications within cells and led to disease pathogenesis.

### State-of-the-art predictors comparison

**Figure 15:**
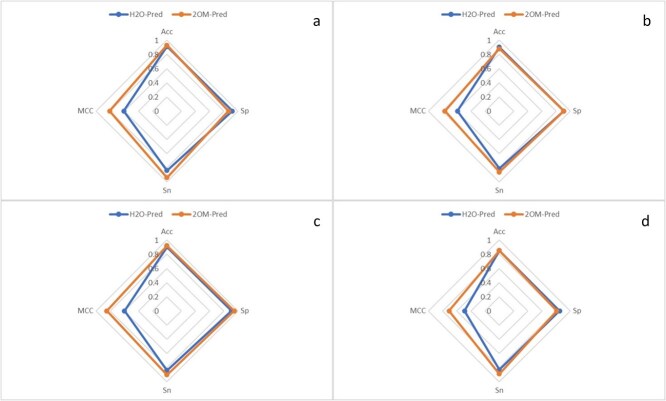
Spider chart of performance comparison of 2OM-Pred with existing predictor.

The performance of the proposed methodology was compared to the preexisting model in terms of accurate predictions. To evaluate the proposed model, comparisons were made with prior research studies, i.e. H2O-Pred, that employed the train-test split method for independent testing. The outcomes are summarized and presented in Table 6. Also, a spider chart has been added to demonstrate the comparative results of 2OM-Pred with the state-of-the-art model, as shown in Fig. 15.

**Table 6:** Comparative analysis of 2OM-Pred with H2O-Pred

Models	A2OM				G2OM				C2OM				U2OM			
	***A***_***CC***_	***S***_***pc***_	***S***_***en***_	***M***_***CC***_	***A***_***CC***_	***S***_***pc***_	***S***_***en***_	***M***_***CC***_	***A***_***CC***_	***S***_***pc***_	***S***_***en***_	***M***_***CC***_	***A***_***CC***_	***S***_***pc***_	***S***_***en***_	***M***_***CC***_
**H2O-Pred**	0.91	**0.92**	0.84	0.61	0.90	0.91	0.84	0.60	0.90	0.91	0.81	0.59	0.85	0.85	0.83	0.49
**2OM-Pred**	**0.93**	0.87	**0.94**	**0.81**	**0.92**	**0.95**	**0.90**	**0.85**	**0.88**	0.91	**0.86**	**0.77**	0.85	0.81	**0.89**	**0.71**

Bold values indicate the higher performance scores between the compared models for each metric.

The proposed model, 2OM-Pred, outperformed in almost all accuracy metrics using independent tests. The H2O-Pred model was selected for comparison in our study because it represents the most recent advancement in the field and has been evaluated using well-established benchmark datasets. The H2O-Pred study incorporated these data sets and provided a detailed comparison with previously developed 2OM site predictors, highlighting its superior performance over those earlier models. However, in our current research, the proposed model, 2OM-Pred, achieved an even higher accuracy score than H2O-Pred, demonstrating notable improvements in prediction performance, as also mentioned in Table 6. Adopting novel feature extraction methods, rigorous training and validation of various computationally intelligent models and speciewise comparison of accuracy scores with H2O-Pred have proved the proposed model, 2OM-Pred, to be superior. Moreover, 2OM-Pred was tuned with a customized training-validation strategy to prevent overfitting and improve generalization, further contributing to the observed performance gains.

This enhancement suggests that 2OM-Pred has effectively addressed limitations observed in previous approaches. Consequently, based on empirical evidence, we assert that our proposed model, 2OM-Pred, significantly outperforms existing 2OM site predictors, including the state-of-the-art H2O-Pred.

The improved prediction accuracy of our model for 2OM sites has important biological implications beyond mere classification performance, specifically in the accurate identification of novel sites and understanding of site-specific regulation. Enhanced predictive power enables discovering of previously undetected 2OM sites across diverse RNA molecules and cell types. This can guide experimental validation efforts, reducing the cost, and labor associated with genome-wide experimental screening. Since 2OM modifications play crucial roles in RNA stability, translation, and immune recognition, precise prediction helps identify regulatory patterns such as sequence motifs and structural preferences.

## Access to webserver

A web-based server presents a practical, user-friendly solution for quick, and convenient computing tasks. In line with this concept, a webserver specifically named 2OM-Pred has been thoughtfully designed for the proposed model. The webserver has been developed using Python library streamlit (version 1.40.0) [44]. The web-based application is then deployed on the Steamlit webserver. The service is free to use, and its primary purpose is to assist with the computations required by the proposed model. Interested users can access the webserver at https://2om-pred-webapp.streamlit.app/.

### Webservers exception

**Figure 16:**
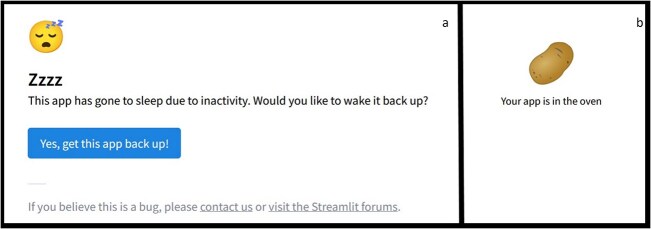
(a) Inactive webserver. (b) Webserver reactivation.

When the webserver applications are active, they perform optimally. However, after a prolonged period of inactivity, the webservers enter hibernation mode. The system displays a dialog window when users click the webserver icon, as shown in Fig. 16. The message informs the user that the webserver is currently turned off. To restart the webserver, users can click "Yes, get this app backup!" The webserver resumes operations within a few seconds after the user selects this option. Figure 16 illustrates what appears on the screen when users perform this retrieval action.

## Conclusion

The current research employed ensemble learning models to predict 2OM, a widespread RNA PTM. RNA data were examined using position-based feature extraction and composition analysis of nucleotides paired with statistical moment techniques to simplify features. The training is performed using boosting and bagging ensemble algorithms to process the dataset for analysis. The models are then tested using cross-validation to validate the authentication of the results. Multiple popular accuracy metrics were used to find the best model. The performance of proposed models on the same accuracy metrics was evaluated compared to pre-existing models. The final recommendation for our solution is the selected bagging ensemble, as its performance outshone the other models in our study. To evaluate 2OM-Pred, it was compared with other available predictors. The results show that 2OM-Pred achieved the highest accuracy among the competing models, indicating that the proposed approach outperforms previous methods for identifying modified 2OM sites.

**Key Points**

- 2-*O*-methylation affects RNA stability and epigenetic regulation.
- Previous methods struggled with RNA modification site identification.
- New feature generation via matrices, vectors, and dimensionality reduction.
- Prediction models were evaluated using *k*-fold cross-validation.
- The bagging ensemble model achieved optimal accuracy in prediction.

## Data Availability

The code and data of the proposed models are accessible at https://github.com/taseersuleman/2OM-Pred.
